# Perceptions of Physical Activity Promotion, Transportation Support, Physical Activity, and Body Mass: an Insight into Parent-Child Dyadic Processes

**DOI:** 10.1007/s12529-019-09780-9

**Published:** 2019-04-08

**Authors:** Karolina Horodyska, Monika Boberska, Magdalena Kruk, Zofia Szczuka, John Wiggers, Luke Wolfenden, Urte Scholz, Theda Radtke, Aleksandra Luszczynska

**Affiliations:** 1 Department of Psychology, SWPS University of Social Sciences and Humanities, 30b Ostrowskiego St, 53238 Wroclaw, Poland; 2 Hunter New England Population Health, Locked Bag 10, Wallsend, Newcastle, NSW 2287 Australia; 3 School of Medicine and Public Health, University of Newcastle, University Drive, Callaghan, Newcastle, NSW 2308 Australia; 4 Hunter Medical Research Institute, Kookaburra Ct, New Lambton, Newcastle, NSW 2300 Australia; 5 Department of Psychology, University of Zurich, Binzmühlestrasse 14, Box 14, 8050 Zurich, Switzerland; 6 Trauma, Health, & Hazards Center, University of Colorado, 1861 Austin Bluffs Pkwy, Colorado Springs, CO 80933-7150 USA

**Keywords:** Childhood obesity, Moderate-to-vigorous physical activity, Health promotion, Instrumental social support

## Abstract

**Background:**

Socio-ecological models indicate that family, school, and community environment explains children’s physical activity and body weight. This study investigated whether parental perceptions of school/community-based physical activity (PA) promotion programs as well as parental and child perceptions of parental instrumental support for child PA (transportation provision) would predict child body weight. Child moderate-to-vigorous physical activity (MVPA) was hypothesized to mediate these associations.

**Method:**

Data of 879 parent-child dyads were collected at two measurement points: the baseline (T1) and the 7–8-month follow-up (T2). Parents were 23–68 years old (83.3% women), while children were 5–11 years old (52.4% girls). Parents and children reported their perceptions of environment, support (T1), and MVPA (T1, T2). Parental and child body weight and height were measured objectively (T1, T2).

**Results:**

Path analyses indicated indirect effects of parental perceptions of school/community-based PA policies (T1) and parental perceptions of transportation provision (T1) on child body weight (T2), with child MVPA (T2) operating as the mediator. There were no direct or indirect effects of child perceptions of parental transportation provision (T1) on child MVPA or body weight (T2). Similar patterns of associations were found for the total sample and the subsample of children with overweight/obesity.

**Conclusion:**

Parental perceptions of school/community-based PA policies and transportation provision may explain changes in child MVPA and body weight. Interventions aimed at prevention of child overweight/obesity may benefit from a focus on parental transportation provision to PA facilities and parental awareness of PA promotion at local environment.

**Electronic supplementary material:**

The online version of this article (10.1007/s12529-019-09780-9) contains supplementary material, which is available to authorized users.

## Introduction

An insufficient physical activity is one of the key determinants of overweight/obesity in children [1]. Children who engage in moderate-to-vigorous physical activity (MVPA) have a reduced risk of non-communicable diseases, including cardiovascular and musculoskeletal diseases [2]. The World Health Organization [3] recommends that children should accumulate at least 60 min of MVPA per day. However, European population surveys found that the percentage of children meeting MVPA guidelines was generally low, ranging from 2.0% to 14.7% in girls and from 9.5% to 34.1% in boys [4]. Moreover, children with overweight/obesity are less active than children with normal body mass [5].

According to the socio-ecological models of health behaviors [6–8], factors operating at multiple levels may influence obesity/overweight-related behavior. These factors include self-perceptions, social and physical environment, as well as policy-related determinants. The socio-ecological model of childhood obesity proposed by Davison and Birch [7] assumes that three groups of factors explain child overweight/obesity. The first group refers to child behaviors (e.g., physical activity (PA), dietary behaviors, and sedentary behaviors). The second group of predictors includes parental PA-related variables, namely parental PA (behavior and behavioral preferences) and parental support for child PA. Finally, the third group of predictors refers to sociodemographic, school, and community characteristics (such as school system and school-based PA policies). In line with Davison and Birch’s model [7], research explaining child overweight/obesity should apply dyadic determinants, that is determinants measured in two members of the dyad (a child and a parent). The present study will investigate the three groups of predictors of child body mass, accounting for PA-related factors included in Davison and Birch’s model [7]. In particular, we will investigate the direct effects of child PA (the first group of predictors), parental PA, parental support for child PA (the second group of predictors), sociodemographic and community characteristics, including parental and child age and gender, parental education and economic status, as well as perceptions of school and community PA-promoting policies (the third group of predictors).

### The Predictive Role of Parental Transportation Support Provision

Parental influence on child behaviors, such as MVPA, is especially strong in children < 12 years old [9, 10]. Parents of young children serve as “gate keepers,” controlling access to PA-promoting environments [11]. As suggested by Davison and Birch [7], parental support is a key parental predictor of child PA and body mass. However, our previous analyses conducted in parent-child dyads showed that an overall PA support reported by parents did not predict the overall PA levels in children with overweight/obesity [12]. However, our previous analyses [12] tested the effects of a global index of parental PA support (combining instrumental, emotional, transportation support) on child PA. Furthermore, child perceptions of parental support were not considered. Using the same dataset (as applied by Liszewska et al., [12]), the present study provides a closer look into the role of a specific type of parental support: transportation provision. Additionally, this study will test the effects of this specific type of support on child body mass at a follow-up, accounting for PA, body mass at the baseline, and support provision assessed in both child and parent.

The social support framework developed by Beets et al. [9] classifies parental transportation provision as a key type of instrumental support for PA. This framework [9] assumes that parental transportation support directly affects child PA. Research confirmed that parental transportation support is associated with child PA [9, 10, 13, 14], fitness among children < 12 years old [15], and changes in child PA over time [16]. Existing evidence, however, has several weaknesses. To date, evidence for associations between child PA and parental transportation support has been gathered mostly in cross-sectional studies [9, 14]. Furthermore, research usually focused on the roles of either parental [17] or children’s perceptions [18], whereas dyadic studies are scarce. To our knowledge, there are only two studies [19, 20] examining the role of both parental and child perceptions of parental transportation support provision. These studies [19, 20] yielded contradictory results.

It is unclear whether the associations between parental transportation support, child MVPA, and child body mass are equivalent (or dissimilar) in children with normal body mass and in children with overweight/obesity. Some studies indicated that children with overweight/obesity and those with normal body mass perceived similar amount of parental support (including transportation provision), whereas other research suggested disparity in parental support, with children with overweight/obesity reporting lower levels of parental support [9]. Thus, this study will investigate if the direct and indirect effects of parental transportation support provision are equivalent in children with normal body mass compared to those with overweight/obesity.

### The Role of Perceived School and Community-Based PA Promotion Programs

School PA policies (e.g., time allowed for free play, time spend outdoors) are among the key environmental determinants of child PA [21]. In turn, community-based PA promotion programs (e.g., city fitness nights targeting families, community fitness programs) are among the key environmental determinants of child body mass and overweight/obesity rates [22]. These environmental determinants have the strongest effects on PA among children < 12 years old [21]. Furthermore, perceptions of school and community-based programs were associated with child body mass and with overweight/obesity-related behaviors [23].

To date, the evidence for the relationships between the school and community-based PA promotion programs and child PA was usually obtained in cross-sectional research [23–25]. Additionally, research usually relied on perceptions of PA school policies reported by either teachers or school administrators [24, 25] or children only [23]. Prospective effects of parental perceptions of PA promotion programs on child body mass (via MVPA) remain unknown. Importantly, the associations between these three variables (perceptions of school and community-based PA promotion programs, child PA, and child body mass) were tested almost solely in cross-sectional studies [25, 26].

### The Aims

Applying a prospective design among parent-child dyads, this study aims to explain child MVPA and child body mass. In particular, we hypothesized that parental perceptions of school and community-based PA promotion programs (reported at the baseline), as well as parental and child perceptions of parental transportation provision (reported at the baseline), would directly predict child MVPA (evaluated at the 7–8-month follow-up). Second, it was hypothesized that these three types of perceptions would indirectly predict child body mass (evaluated at the 7–8-month follow-up), with child MVPA (evaluated at the 7–8-month follow-up) acting as the mediator. These associations were hypothesized for the total sample. Additionally, we investigated if associations assumed in the hypothesized model (including both direct and indirect effects) would be equivalent across two subgroups: (1) dyads with children with normal body mass and (2) dyads with children with overweight/obesity.

## Methods

### Participants

This study was a part of a larger prospective correlational project, investigating dyadic predictors of PA and body mass indicators in parent-child dyads. For more details, see Horodyska et al. [27] and Liszewska et al. [12].

Dyads of children (aged 5–11) and their parents (the mother, father, or a legal guardian) were invited to participate in the study. The parent (or legal guardian) who declared that she/he was the main person organizing daily activities of the child (including child PA) was invited to participate in the study. Participants were enrolled in schools and general practitioners’ offices in 26 villages, towns, and cities.

A total of 1000 parent-child dyads were invited to participate. At time 1 (T1), 879 dyads (1758 individuals) provided informed consents; all dyads that provided their consent took part in T1 measurement. At time 2 (T2; 7–8-month follow-up), data from 603 parent-child dyads was collected. Data from the total sample of 879 dyads was analyzed.

### Procedure

The prospective dyadic design was applied. Data were collected from 2011 through 2015, in six regions of Poland (representing 36.3% of the area of the country). In each location (*N* = 26), the research team visited primary schools providing education for children aged 5–11. The team also visited nurse/general practitioners’ offices (conducting routine check-ups in children aged 5–11 years old) and discussed the possibility of data collection in a respective location. Two schools (out of 27) and two practitioners’ offices (out of 12) did not agree to contribute to data collection. Overall, data were collected in *N* = 35 schools or general practitioners’ offices.

After arriving at data collection location, each parent and each child filled in the questionnaires, referring to parental and child perceptions (including PA promotion programs, parental support, other beliefs and perceptions measured in the larger project, cf. [27]) and MVPA. Younger children were interviewed. Next, body mass was measured. All data were collected individually; children and parents provided their data separately.

Time 2 data were collected at 7 to 8 months after T1. The first attempt to collect T2 data took place at 7 months after T1. Attempts to contact participants and collect T2 data were continued for 1 month (representing the eighth month of data collection). The time gap between the measurement points was designed to fit the school year, accounting for an additional month at the beginning of the study to discuss the enrolment with schools’ principals and parent-teacher associations. The time span was chosen to avoid a dropout due to school change after the completion of the school year. T2 measurement procedures were the same as T1 procedures.

Details referring to study personnel, parental and child data collection procedures, and locations are presented in Horodyska et al. [27]. A total of 43 dyads from the sample of *N* = 922 reported by Horodyska et al. [27] were excluded. At this stage, we detected cases which may be considered a passive decline (either a child or a parent provided invalid responses at T1 (e.g., circling a response value of “3” when responding to all policy and support perception questions). To identify a passive decline, data were coded independently by three co-investigators (KH, MB, AL). The inter-rater concordance coefficient for identifying a passive decline was *κ* = 1.00, *p* < .001.

The study was approved by the Internal Review Board at the first author’s institution.

### Measures

The association between the parental and child variables may be influenced by differences in the assessment format. Therefore, the use of the same measures, where applicable, is the optimal solution [28]. A qualitative pilot study with *N* = 18 children (5–11 years old) was conducted. Children were asked to explain the items in their own words and to indicate any phrases they do not understand/are unsure of. The pilot study indicated that younger children reported that they are unsure about the presence/absence of school and community-based programs; therefore, this measure was not applied in children.

Parents reported their perceptions of school- or community-based PA promotion programs at T1. Two items were applied: “At my child’s school, a lot of attention is paid to physical activity promotion for children” and “In our local community, much is being done to help me and my children to be physically active.” The items were based on a measure by Stok et al. [23]. Responses were given on four-item scales ranging from 1 (“definitely not”) to 4 (“exactly true”). The reliability of the measure was acceptable with *α* of 71, *p* < .001, and Spearman-Brown coefficient of .71, *p* < .001.

Parental perceptions of parental PA support (transportation provision) was measured at T1 with one item, “I drive or transport my child (e.g., by bus, car, etc.) to places where he/she can exercise or play sports” [10]. The responses were given on a four-item response scale ranging from 1 (“definitely not”) to 4 (“exactly true”).

Child perceptions of parental instrumental support for child PA (transportation provision) were measured at T1 with one item, “My mom drives me (or takes me by bus or tram) to places where I can exercise or play sports,” based on Edwardson and Gorely [10]. Items were adjusted to the gender of participating parent. The responses were given on four-item response scales ranging from 1 (“definitely not”) to 4 (“exactly true”).

Moderate-to-vigorous physical activity of parents and children was measured at T1 and T2 with two items derived from self-report physical activity questionnaire by Godin and Shephard [29]. Participants were asked to consider a 7-day period (a week) and report how many times they did the following kinds of exercise for more than 15 min during their free time. The first item referred to “strenuous exercise (heart beats rapidly), i.e., running, jogging, hockey, soccer, basketball, judo, roller skating, vigorous swimming, vigorous long distance bicycling,” whereas the second item referred to “moderate exercise (not exhausting), i.e., fast walking, easy bicycling, easy swimming, dancing”. This measure’s validity and reliability were found to be acceptable in studies involving children aged 7–15 [30] and adults [29]. To obtain a total metabolic equivalent (MET) score, the vigorous PA score was multiplied by 9, the moderate PA score was multiplied by 5, and these two scores were summed [29]. Reliability for this two-item measure was moderate with *α* of .55 (T1) and .53 (T2) in children and *α* of .53 (T1) and .59 (T2) in parents and with Spearman-Brown coefficient of .56 (T1) and .53 (T2) in children and *α* of .54 (T1) and .60 (T2) in parents.

Body weight and height of parents and children (T1 and T2) were measured with certified body weight floor scales (BF-100 and BF-25; Beurer, Germany, measurement error < 5%) and medically approved telescopic height measuring rods. Parental body mass index (BMI) was calculated at T1 and T2 using parental body weight and height. Values of BMI of 25 or above were coded as indicating overweight or obesity. Child body mass at T1 and T2 was estimated using BMI *z* scores [31].

Sociodemographic characteristics were assessed at T1. The measures accounted for parental and child gender and age, parental education (with responses ranging from primary, uncompleted secondary/vocational, secondary, at least 3 years of higher education, to five or more years of higher education), and parental reports of perceived economic situation (“Compared to the average economic situation of families in the country, how would you rate the economic situation of your family” with responses ranging from 1 “much below the average” to 5 “much above the average”).

### Data Analysis

GPower calculator [32] was used to calculate the sample size. We assumed small effect sizes (*f*^2^ = .03) of predictor variables on child PA and body mass. Besides the main predictors, analyses accounted for potential confounders, such as parental and child gender and age, parental education, and perceived economic status. It was estimated that in case of a one-group model, the total sample should include at least 624 dyads. For the two-group model, we estimated that the sample should include at least 806 dyads.

BMI *z* scores, indicating child body mass, were calculated with the data of child body mass and height. The scores were calculated based on WHO growth references, using SPSS macro provided by WHO [31].

Path analyses [33] were conducted with IBM AMOS 25, maximum likelihood estimation. Missing data (including data missing due to dropouts at T2) were accounted for using full information maximum likelihood imputation procedure (FIML, [33]). Multivariate normality was checked with Mardia’s coefficient, with a value of 25.20 for the one-group model and 21.81 for the two-group model, indicating moderate non-normality. Several model-data fit indices were applied. We used a cut-off point < .08 for root mean square error of approximation (RMSEA) and standardized root mean residual (SRMR). A cut-off point > .90 was applied for comparative fit index (CFI), Tucker-Lewis index (TLI), goodness-of-fit index (GFI), and normed fit index (NFI) [33]. The significance of indirect effects was evaluated with unstandardized effect coefficients, after applying a bootstrapping method (10,000 bootstraps; 90% confidence intervals; models were re-run with 5000 bootstraps and 95% confidence intervals to check the stability of the patterns).

The path analysis tested the associations in the hypothesized model for the total sample. The hypothesized associations were investigated controlling for the baseline parental and child gender and age, parental education, and perceived economic status (reported by parents). Next, we analyzed the two-group model, including (1) a subsample of dyads with children of normal body mass and (2) a subsample of dyads with children with overweight/obesity. The unconstrained two-group model was compared with the nested models specifying model invariance across both subsamples. The constrained model should be accepted if there is no significant difference between the unconstrained and nested models and if the changes of model-data fit (ΔTLI) are small [33].

To account for non-independence, the respective predictors measured in the dyad members (e.g., parental and child MVPA at T1) were allowed to covary. In line with the Actor Partner Interdependence Model [34], the hypothesized and nested models accounted for covariations between respective child and parent variables at respective measurement points. Covariances between the controlled variables and the main study variables were included.

**Fig. 1:**
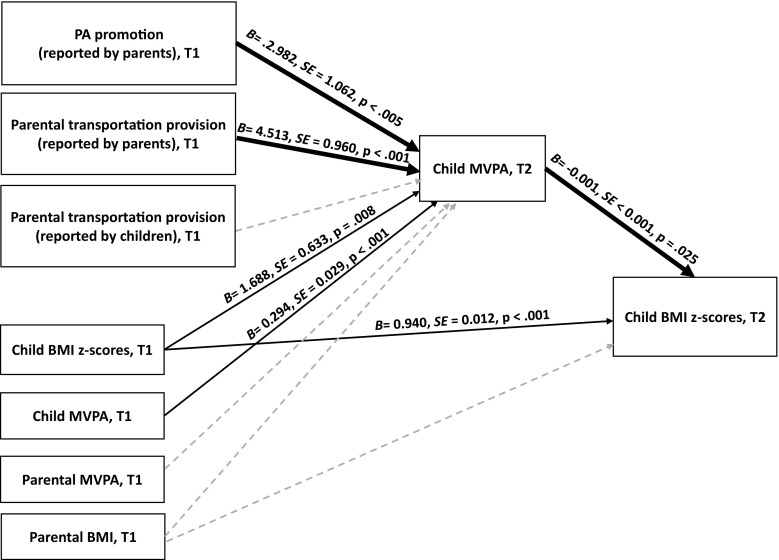
Results of path analysis: the hypothesized model for the total sample (*n* = 879). T1, time 1 (the baseline); T2, time 2 (the 7–8-month follow-up). The values of unstandardized path coefficients, SE, and *p* levels are displayed only for significant coefficients. Solid lines represent path coefficients which were significant. Bold solid lines represent significant indirect effects. Dashed lines represent path coefficients which were not significant. For clarity, the covariances were not displayed. All path coefficients, covariance coefficients, and bias-corrected confidence intervals (90%) for path coefficients are presented in Table 1

The hypothesized effects for the total sample (one-group model) are presented in Fig. 1. For clarity, the figure does not display covariances between T1 variables, effects of age, gender, education, and economic status. The hypothesized effects for the two-group model (the subsample with children with normal body mass vs. the subsample with children with overweight/obesity) are presented in Table 1.

**Table 1:** Regression coefficients and covariances for the study variables

Variable	The total sample			Children with normal body mass			Children with overweight/obesity		
Path coefficients/covariance coefficients	Estimate (90% BCI)	SE	*p* value	Estimate (90% BCI)	SE	*p* value	Estimate (90% BCI)	SE	*p* value
Predictors of child MVPA (T2)									
PA promotion (P, T1) → MVPA (Ch, T2)	**2.982 (1.290, 4.662)**	**1.062**	**.005**	**2.605 (0.597, 4.586)**	**1.326**	**.050**	3.318 (− 0.087, 6.859)	2.049	.105
Transportation (P, T1) → MVPA (Ch, T2)	**4.513 (2.696, 6.456)**	**0.960**	**< .001**	**3.928 (1.735, 6.433)**	**1.206**	**< .001**	**5.428 (1.640, 9.279)**	**1.930**	**.005**
Transportation (Ch, T1) → MVPA (Ch, T2)	0.024 (− 1.819, 1.633)	0.799	.976	**−** 0.214 (− 2.633, 1.765)	1.003	.831	0.242 (− 2.906, 3.454)	1.556	.876
BMI (P, T1) → MVPA (Ch, T2)	0.134 (− 0.210, 0.486)	0.213	.530	0.143 (− 0.292, 0.570)	0.279	.608	0.278 (− 0.380, 0.886)	0.369	.451
BMI *z* scores (Ch, T1) → MVPA (Ch, T2)	**1.688 (0.658, 2.676)**	**0.633**	**.008**	− 0.219 (− 2.835, 2.129)	1.426	.878	2.248 (− 1.058, 5.582)	2.290	.326
MVPA (P, T1) → MVPA (Ch, T2)	0.018 (− 0.042, 0.077)	0.041	.664	0.067 (− 0.014, 0.142)	0.052	.200	**−** 0.053 (− 0.181, 0.073)	0.075	.476
MVPA (Ch, T1) → MVPA (Ch, T2)	**0.294 (0.229, 0.364)**	**0.029**	**< .001**	**0.349 (0.266, 0.441)**	**0.036**	**< .001**	**0.134 (0.021, 0.260)**	**0.057**	**.018**
Gender (P, T1) → MVPA (Ch, T2)	1.795	2.150	.404	3.503	2.794	.210	**−** 2.959	3.858	.443
Gender (Ch, T1) → MVPA (Ch, T2)	− **3.540**	**1.544**	**.022**	**−** 3.116	1.937	.108	**− 6.419**	**2.967**	**.030**
Age (P, T1) → MVPA (Ch, T2)	0.198	0.129	.123	0.279	0.165	.090	**−** 0.376	0.249	.131
Age (Ch, T1) → MVPA (Ch, T2)	0.735	0.577	.203	0.788	0.721	.274	0.412	1.197	.731
Education (P, T1) → MVPA (Ch, T2)	**−** 1.231	0.669	.066	**−** 1.057	0.836	.207	**−** 0.473	1.290	.714
Economic status (P, T1) → MVPA (Ch, T2)	**−** 0.761	0.975	.435	**−** 0.493	1.227	.688	**−** 1.939	1.825	.288
Predictors of child *z* BMI score (T2)									
MVPA (Ch, T2) → BMI *z* scores (Ch, T2)	− **0.001 (− 0.002, - 0.001)**	**< 0.001**	**.025**	**−** 0.001 (− 0.002, 0.001)	0.001	.163	− **0.003 (− 0.005, − 0.001)**	**0.001**	**< .001**
BMI (P, T1) → BMI *z* scores (Ch, T2)	0.001 (− 0.005, 0.008)	0.004	.742	0.002 (− 0.008, 0.012)	0.005	.718	0.002 (− 0.007, 0.010)	0.005	.705
BMI *z* scores (Ch, T1) → BMI *z* scores (Ch, T2)	**0.940 (0.922, 0.958)**	**0.012**	**< .001**	**0.929 (0.886, 0.972)**	**0.027**	**< .001**	**0.921 (0.875, 0.958)**	**0.031**	**< .001**
Gender (P, T1) → BMI *z* scores (Ch, T2)	0.029	0.040	.467	− 0.009	0.054	.862	0.018	0.053	.735
Gender (Ch, T1) → BMI *z* scores (Ch, T2)	**−** 0.005	0.029	.867	0.013	0.037	.722	− 0.044	0.041	.286
Age (P, T1) → BMI z-scores (Ch, T2)	**−** 0.001	0.002	.716	< .001	0.003	.916	− 0.002	0.003	.538
Age (Ch, T1) → BMI z-scores (Ch, T2)	**−**0.010	0.011	.348	**−** 0.009	0.014	.520	− 0.008	0.016	.609
Education (P, T1) → BMI z-scores (Ch, T2)	**0.025**	**0.012**	**.031**	0.027	0.015	.085	0.025	0.016	.135
Economic status (P, T1) → BMI z-scores (Ch, T2)	**−** 0.007	0.018	.698	**−** 0.011	0.023	.626	0.039	0.025	.119
Covariances									
PA promotion (P, T1) ←→ MVPA (Ch, T1)	0.010	0.657	.988	0.011	0.836	.990	0.203	1.302	.876
PA promotion (P, T1) ←→ Transportation (P, T1)	0.039	0.024	.099	**0.069**	**0.030**	**.022**	− 0.007	0.048	.887
PA promotion (P, T1) ←→ Transportation (Ch, T1)	0.026	0.027	.335	0.003	0.035	.941	0.062	0.055	.265
PA promotion (P, T1) ←→ Economic status (P, T1)	0.014	0.021	.507	0.007	0.026	.778	**−** 0.003	0.042	.950
PA promotion (P, T1) ←→ Education (P, T1)	**−** 0.050	0.031	.109	**−** 0.048	0.039	.213	**−** 0.073	0.064	.254
Transportation (P, T1) ←→ MVPA (Ch, T1)	**2.871**	**0.817**	**< .001**	**2.020**	**1.035**	**.051**	**4.466**	**1.620**	**.006**
Transportation (P, T1) ←→ Transportation (Ch, T1)	**0.508**	**0.040**	**< .001**	**0.531**	**0.051**	**< .001**	**0.563**	**0.083**	**< .001**
Transportation (P, T1) ←→ Economic status (P, T1)	**0.181**	**0.028**	**< .001**	**0.194**	**0.035**	**< .001**	**0.187**	**0.057**	**< .001**
Transportation (P, T1) ←→ Education (P, T1)	**0.404**	**0.043**	**< .001**	**0.380**	**0.053**	**< .001**	**0.483**	**0.090**	**< .001**
Transportation (Ch, T1) ←→ MVPA (Ch, T1)	**6.085**	**1.008**	**< .001**	**6.374**	**1.285**	**< .001**	**4.688**	**1.998**	**.019**
Transportation (Ch, T1) ←→ Economic status (P, T1)	**0.140**	**0.031**	**< .001**	**0.160**	**0.039**	**< .001**	0.084	0.065	.195
Transportation (Ch, T1) ←→ Education (P, T1)	**0.239**	**0.047**	**< .001**	**0.216**	**0.059**	**< .001**	**0.301**	**0.099**	**.002**
MVPA (P, T1) ←→ BMI (P, T1)	− **6.587**	**2.428**	**.007**	**−** 5.183	2.784	.063	− **15.025**	**5.814**	**.010**
MVPA (P, T1) ←→ BMI *z* scores (Ch, T1)	**−** 0.097	0.809	.905	0.062	0.542	.909	**−** 0.823	0.908	.365
MVPA (Ch, T1) ←→ MVPA (P, T1)	**97.787**	**17.680**	**< .001**	**94.058**	**21.797**	**< .001**	**122.494**	**38.382**	**.001**
MVPA (Ch, T1) ←→ BMI (P, T1)	0.174	3.339	.959	**−** 1.910	−0.384	.656	2.153	7.373	.770
MVPA (Ch, T1) ←→ BMI *z* scores (Ch, T1)	**−** 0.264	1.118	.814	**−** 0.882	0.971	.358	0.019	1.168	.987
BMI (P, T1) ←→ BMI *z* scores (Ch, T1)	**0.901**	**0.159**	**< .001**	**0.273**	**0.101**	**.007**	− 0.823	0.908	.365
BMI (P, T1) ←→ Age (P, T1)	**3.796**	**0.766**	**< .001**	**3.910**	**0.896**	**< .001**	**4.384**	**1.690**	**.009**
BMI (P, T1) ←→Gender (P, T1)	**− 0.390**	**0.049**	**< .001**	− **0.368**	**0.057**	**< .001**	− **0.411**	**0.114**	**< .001**
Economic status (P, T1) ←→ Education (P, T1)	**0.324**	**0.038**	**< .001**	**0.302**	**0.046**	**< .001**	**0.329**	**0.078**	**< .001**

Significant coefficients are marked in bold

*MVPA* moderate-to-vigorous physical activity, *P* parent, *Ch* child, *T1* time 1, *T2* time 2 (7–8-month follow-up), *PA promotion* parental perceptions of school- or community-based PA promotion programs, *Transportation* parental or child perceptions of parental instrumental support for child PA (transportation provision). Significant coefficients are marked in bold

Additional analyses were conducted to explore the patterns of associations in three two-group models: (1) mother-child vs. father-child dyads, (2) parent-daughter vs. parent-son dyads, and (3) dyads with a parent with normal body mass vs. dyads with a parent with overweight/obesity. The results of these analyses are reported in Additional file 1.

## Results

Children were 5–11 years old (*M* = 8.46, SD = 1.34), with 52.4% girls and 47.6% boys. A total of 65.5% (*n* = 576) of children had a normal body mass, whereas 24.3% (*n* = 214) of children were overweight/obese and 10.1% (*n* = 89) were underweight when applying the International Obesity Task Force Thresholds for Excessive Weight [35].

Parents (*N* = 879, 16.7% men, 83.3% women) were 23 to 68 years old (*M* = 36.63, SD = 6.09). The majority of parents (59.4%) had a normal body mass, 2% had BMI below 18.50, and 38.5% of parents had a BMI > 25, indicative of overweight or obesity. The majority of parents (41%) had either a secondary education or higher education (40%), whereas 19% declared primary education only. Sixty-one percent of parents reported that they were employed full-time; 23% declared no current employment or being retired. The remaining 16% reported that they were employed part-time. Data referring to economic status and living in urban/rural areas are presented in Horodyska et al. [27].

Descriptive statistics for all measures are reported in Additional file 1.

Attrition analyses indicated that parents who completed T1 and T2 did not differ from dropouts in terms of age (*F* (1, 877) = 3.03, *p* = .08), gender (*χ*^2^ (1, *N* = 879) = 7.46, *p* = .11), BMI (*F* (1, 877) = .12, *p* = .73), and perceived economic status (*F* (1, 877) = .19, *p* = .67). There was a difference in education (*F* (1, 877) = 7.32, *p* = .01). Parents with lower education were more likely to drop out at T2. There were no significant differences in parental perceptions of school- or community-based PA promotion programs (*F* (1, 877) = .36, *p* = .55), parental perceptions of transportation provision (*F* (1, 877) = .01, *p* = .94), and parental MVPA (*F* (1, 877) = .06, *p* = .82). Children who completed T1 and T2 did not differ from dropouts in terms of age (*F* (1, 877) = .78, *p* = .38), gender (*χ*^2^ (1, *N* = 879) = .83, *p* = .36), perceptions of parental transportation provision (*F* (1, 877) = .30, *p* = .59), and T1 MVPA (*F* (1, 877) = .04, *p* = .83). There was a difference in child BMI *z* scores at T1 (*F* (1, 877) = 9.90, *p* = .002); children with higher BMI *z* scores were more likely to drop out at T2.

Child MVPA did not change over time (*F* (1, 878) = 3.87, *p* = .05. *η*^2^ = .004). Child BMI *z* scores were higher at T1 (*M* = .44; SD = 1.24) than at T2 (*M* = .30; SD = 1.24) (*F* (1, 878) = 88.41, *p* < .001, *η*^2^ = .091). Correlations between the study variables are presented in Additional file 1.

### Effects of Parental and Child Perceptions (T1) on Child MVPA and BMI *Z* Scores (T2): Results for the Total Sample

The hypothesized model, calculated for the total sample (*N* = 879 dyads), presented an acceptable fit (with *χ*^2^ (62) = 231.513, *p* < .001, *χ*^2^/*df* = 3.73, GFI = .966, NFI = .922, TLI = .900, CFI = .941, RMSEA = .056, 90% CI [.048, .064], SRMR = .051). The unstandardized solution is presented in Table 1 (see also Fig. 1). The variables in the hypothesized model explained 88% variance of child BMI *z* scores (T2) and 17% of variance of MVPA (T2).

Parental perceptions of PA promotion programs (T1) directly predicted a higher level of child MVPA (T2). Furthermore, parental perceptions of parental transportation provision (T1) directly predicted a higher level of child MVPA (T2). There were no direct effects of child perceptions of parental transportation provision (T1) on child MVPA (T2).

In accordance with our main hypothesis, child MVPA (T2) mediated the relationship between parental perceptions of PA promotion programs (T1) and child BMI *z* scores (T2), with an indirect coefficient (unstandardized value) of *B* = − .004, 90% BCI [− .009, − .001]. Furthermore, child MVPA (T2) mediated the relationship between parental perceptions of parental transportation provision (T1) and child BMI *z* scores (T2), with an indirect coefficient (unstandardized value) of *B* = − .006, 90% BCI [− .012, − .002]. There were no indirect effects of child perceptions of parental transportation provision (T1) on child BMI *z* scores (T2), with child MVPA (T2) operating as the mediator.

### Results of Path Analysis with a Two-Group Model: Children with Normal Body Mass vs. Those with Overweight/Obesity

We tested if the patterns of associations were similar across two groups: dyads with children with normal body mass (65.5%, *n* = 576) and dyads with children with overweight/obesity (24.3%, *n* = 214). The model fit for the two-group unconstrained model was acceptable (with *χ*^2^ (124) = 291.015, *p* < .001, *χ*^2^/*df* = 2.347, GFI = .954, NFI = .859, TLI = .847, CFI = .910, RMSEA = .041, 90% CI [.035, .048], SRMR = .055). The unstandardized path coefficients for the unconstrained model for the subsample of children with normal body mass and with children with overweight/obesity are reported in Table 1.

We compared the unconstrained two-group model and the constrained two-group models, assuming that structural covariances and residuals are equal across the groups. The comparison indicated that covariances and residuals are similar across the groups (covariances equal: ΔTLI = − .045, *p* = .326 for Δ*χ*^2^; residuals equal: ΔTLI = − .018, *p* = < .01 for Δ*χ*^2^).

The findings for the subgroup of dyads with children with normal body mass showed no indirect effects of parental or child perceptions on child BMI *z* scores (T2), through the mediator, child MVPA (T2). Variables in the equation explained 67% variance of child BMI *z* scores (T2) and 19% variance of child MVPA (T2).

The findings for the subgroup of dyads with children with overweight/obesity showed that there were two significant indirect effects: child MVPA (T2) mediated the relationship between parental perceptions of PA promotion programs (T1) and child BMI *z* scores (T2), with the indirect coefficient (unstandardized values) of *B* = − .010, 90% BCI [− .026, − .001]. Furthermore, child MVPA (T2) mediated the relationship between parental perceptions of parental transportation provision (T1) and child BMI *z* scores (T2), with the indirect coefficient (unstandardized value) of *B* = − .016, 90% BCI [− .037, − .004]. There was no indirect effect of child perceptions of parental transportation provision (T1) on child body mass (T2). Variables in the equation explained 79% variance of child BMI *z* scores (T2) and 15% variance of child MVPA (T2).

Additional analyses explored if the indirect effects may be similar in three two-group models, comparing (1) mother-child dyads vs. father-child dyads, (2) parent-daughter dyads vs. parent-son dyads, and (3) dyads with parents with normal body weight vs. dyads with parents with overweight/obesity. The findings are reported in Additional file 1. Overall, the analyses for the three models assuming that the indirect effects were similar across the two groups yielded acceptable model-data fit. Across groups, the observed patterns of associations were similar to the patterns found in the total sample.

## Discussion

The findings partially support the hypothesized model. In particular, we found indirect effects of parental perceptions of PA policies and parental perceptions of transportation provision on child body mass (through child MVPA). These effects occurred in the total sample and in the subsample of dyads with children with overweight/obesity. In contrast to our hypothesis, there were no effects of child perceptions. Additionally, direct effects of parental perceptions on child MVPA were observed, Parental (but not child’s) perceptions of parental transportation support provision (measured at the baseline) explained a higher level of MVPA in children (at 7–8 months follow-up) in the total sample, the subsamples of dyads with children with normal weight and with children with overweight/obesity. Furthermore, parental perceptions of PA promotion programs (measured at the baseline) explained a higher level of child MVPA at the follow-up (effects found in the total sample and in the subsample with children with normal body weight).

Although parental instrumental support for child PA was often studied [9, 10, 13, 14], previous studies on parental transportation provision were mostly cross-sectional and focused on either parental perceptions only [17] or child perceptions only [18]. Our study goes beyond previous research as it applied a dyadic approach to test whether parental or child perceptions of parental transportation provision may explain child MVPA and, indirectly, lower child BMI *z* scores at a follow-up.

The results indicating significant effects of parental (but not child) perceptions of transportation provision are in line with the invisible support hypothesis [36]. This hypothesis suggests that the provision of support which is unnoticed by the support recipient is assumed to be the most effective [36]. It is also possible that if support is visible, children may perceive that their behavior is being regulated or controlled by parents, which in turn may promote resistance against supported behavior [37]. So far, research on invisible support focused on romantic couples and identified invisible support as a difference score between provider and recipient’s reports. Our approach, testing the effects of both support indicators accounting for covariance between them, may represent another way to investigate the invisible support hypothesis.

The study found that parental reports of high PA promotion programs predicted higher levels of child MVPA (the mediator) that in turn explained lower body mass in children. Such findings extend previous research which did not account for a parental perspective or dyadic indicators [24, 25]. Parental awareness of programs promoting PA in the local environment may be one of the key conditions of successful implementation conditions and vectors of the effectiveness of PA promotion programs [38]. Programs designed to prevent child overweight/obesity may aim at enhancing parental awareness of PA promotion.

Two previous investigations used the same dataset, obtained in a larger longitudinal project [12, 27]. In particular, the study [12] accounting for parental (but not child) reports of PA-enhancing strategies showed that the general indicator of parental support (accounting for instrumental, informational, and emotional support) did not predict PA among children with overweight/obesity. To explain this not significant effect, we re-investigated these associations in the present study, accounting for dyadic processes and a specific type of instrumental support, in an attempt to explain child PA and, additionally, child body mass. In consequence, the present study yielded new evidence, pointing to the role of a specific type of support (transportation provision) in predicting child body mass. The present analyses and those conducted by Horodyska et al. [27] consistently showed that child perceptions did not play a predictive role when explaining obesity indicators.

The strength of associations between the two parental perceptions (PA promotion and transportation provision) and z-BMI in children was low. However, as the baseline measure of z-BMI explained 88% of the variance of z-BMI at T2, the remaining variables in the equation could produce only small or very small effects. It has to be noted that the time span between the measurement of parental perceptions (T1) and the outcome variable (z-BMI; T2) was relatively long, which further reduces the effect sizes. The estimation of the clinical relevance of the direct and indirect effects of the two significant predictors observed in the overall model should be confirmed in an experimental study, testing if an intervention addressing these two predictors may result in clinically significant changes of body mass, particularly among children with overweight/obesity.

Findings from our study may have implications for child overweight/obesity prevention. Parental involvement in prevention and treatment is a well-established condition of effectiveness of such programs [39]. Interventions and education programs may aim at enabling parents to effectively support their children to be active. Parents should be made aware of how their actions, such as transportation provision to places where children could be active, may influence children’s participation in MVPA and childhood overweight/obesity.

The present study has several limitations. The reliability and validity of MVPA may be considered acceptable (but relatively low) in samples consisting of young children [30]. Therefore, the findings need to be treated with caution. Due to a large sample size (879 parent-child dyads), accelerometer-based measurement of MVPA was not feasible. The time gap between the measurement points was relatively short (7–8 months). It was chosen to reduce the likelihood of dropout due to school change after the completion of the school year. The time gap of 7–8 months between the measurement points contributed to limited variability of body mass. The significant associations between parental perceptions of transportation provision and child PA, which was found in Forthofer et al. [20] and in our study, may be specific for children younger than 12 years old. In general, parental variables are stronger predictors of child PA, compared to PA of adolescents [27]. Age-specific effects of perceived transportation provision may require more thorough investigation. The measure of perceived provision of transportation support did not account for the need of such support. In other words, low scores of support provision may mean that (1) respective support was not provided because it was not needed (e.g., there was an abundance of PA facilities in a proximal environment) or (2) there was a need, but support was not provided. Future research should account for the need of support provision, perceived by parents and children. Next, the measure of transportation support referred mostly to passive means of transport (e.g., traveling by bus). Future studies need to distinguish between the effects of provision of transportation support by active (e.g., cycling) vs. passive (e.g., driving) means. A further limitation is that some predictors were assessed with single items, to keep the participants’ burden low. However, there is evidence that the single items may serve as valid and useful measures [40]. Experimental research conducted among adolescents showed that interventions targeting individual’s beliefs predicted a reduction of body mass, but this effect may depend on the actual presence of built PA facilities in the local community/school environment [41]. Therefore, future research explaining overweight/obesity among young people may need to address individual-level predictors combined with a socio-ecological model [41].

## Conclusions

Our findings provide novel evidence for the associations between parental and child perceptions of transportation provision, parental perceptions of school- or community-based PA promotion programs, child MVPA, and child body mass. Child perceptions of parental transportation support did not explain child outcomes. In contrast, parental perceptions of transportation provision and PA promotion programs predicted child MVPA and body mass measured at 7–8-month follow-up. These effects were found in the total sample and in the subsample of dyads with children with overweight/obesity.

## Electronic supplementary material

ESM 1 (DOCX 60 kb)

## Abbreviations

BMI: Body mass index
PA: Physical activity
MVPA: Moderate-to-vigorous physical activity
T1: Time 1
T2: Time 2
P: Parent
Ch: Child
FIML: Full information maximum likelihood
RMSEA: Root mean square error of approximation
SRMR: Standardized root mean residual
CFI: Comparative fit index
TLI: Tucker-Lewis index
GFI: Goodness-of-fit index
NFI: Normed fit index
BCI: Bootstrap confidence intervals
