# Supplementary material for: Perceptions of Physical Activity Promotion, Transportation Support, Physical Activity, and Body Mass: an Insight into Parent-Child Dyadic Processes
Source: Int J Behav Med. 2019 Apr 8;26(3):255–65. doi: 10.1007/s12529-019-09780-9 (PMC6529394; doi:10.1007/s12529-019-09780-9)
Supplement: Supplementary file 1 — (DOCX 60 kb) [file 12529_2019_9780_MOESM1_ESM.docx]

**Additional File 1**

**Perceptions of physical activity promotion, transportation support, physical activity,**

**and body mass: an insight into parent-child dyadic processes**

The Additional File 1 includes results of additional analyses. Correlations between the study variables for the total sample as well as the descriptive statistics for the total sample are presented in Table 2.

Next, the Additional File 1 reports results of path analysis was applied to explore the three two-group models. We investigated if patterns of associations observed in the total sample are similar to the patterns of associations observed across subgroups of dyads: (1) mother-child dyads in comparison to father-child dyads; (2) parent-daughter dyads in comparison to parent-son dyads and (3) dyads with parents with normal body weight in comparison to dyads with parents with overweight/obesity.

**Table 2.** *Correlations and descriptive statistics for the study variables.*

| Variable | 2 | 3 | 4 | 5 | 6 | 7 | 8 | 9 | 10 | 11 | 12 | 13 | 14 | 15 | 16 | 17 | *M (SD)* |
| --- | --- | --- | --- | --- | --- | --- | --- | --- | --- | --- | --- | --- | --- | --- | --- | --- | --- |
| 1. PA promotion (P, T1) | .06 | .03 | .02 | -.01 | .09 | -.08 | .10 | -.01 | .13 | -.06 | -.03 | -.06 | -.05 | .03 | .02 | -.05 | 2.72 (0.73) |
| 2. Transportation (P, T1) |  | .**64** | .15 | -.06 | .06 | .01 | .21 | -.02 | .05 | .02 | .04 | -.12 | .16 | -.04 | .23 | .34 | 2.98 (0.96) |
| 3. Transportation (Ch, T1) |  |  | .22 | -.01 | .04 | -.00 | .15 | .00 | .03 | .01 | .04 | -.08 | .04 | -.05 | .17 | .18 | 2.73 (1.12) |
| 4. MVPA (Ch, T1) |  |  |  | -.01 | **.32** | .00 | .36 | .01 | .12 | .01 | .05 | -.12 | .03 | .02 | .09 | .25 | 44.67 (27.33) |
| 5. BMI z-scores (Ch, 1) |  |  |  |  | -.01 | **.18** | .08 | .94 | .02 | .16 | .08 | -.04 | -.10 | .01 | .01 | -.06 | 0.44 (1.24) |
| 6. MVPA (P, T1) |  |  |  |  |  | -.08 | .10 | -.02 | .65 | -.09 | -.01 | -.04 | -.03 | -.03 | -.02 | -.10 | 21.93 (19.32) |
| 7. BMI (P, 1) |  |  |  |  |  |  | .04 | .16 | -.05 | .98 | .11 | -.02 | .19 | -.29 | -.09 | -.04 | 24.44 (3.92) |
| 8. MVPA (Ch, T2) |  |  |  |  |  |  |  | .05 | **.29** | .04 | .08 | -.14 | .07 | .01 | .03 | -.01 | 46.66 (25.28) |
| 9. BMI z-scores (Ch, 2) |  |  |  |  |  |  |  |  | .01 | **.16** | .07 | -.04 | -.10 | .01 | .01 | -.03 | 0.30 (1.24) |
| 10. MVPA (P, T2) |  |  |  |  |  |  |  |  |  | -.06 | -.01 | -.04 | -.04 | -.04 | -.03 | -.11 | 22.14 (17.24) |
| 11. BMI (P, 2) |  |  |  |  |  |  |  |  |  |  | .10 | -.02 | .18 | -.29 | -.09 | -.03 | 24.45 (3.95) |
| 12. Age (Ch) |  |  |  |  |  |  |  |  |  |  |  | -.01 | **.15** | -.08 | .01 | -.01 | 8.46 (1.34) |
| 13. Gender (Ch) |  |  |  |  |  |  |  |  |  |  |  |  | .00 | .03 | -.04 | -.03 |  |
| 14. Age (P) |  |  |  |  |  |  |  |  |  |  |  |  |  | -.19 | .07 | .14 | 36.64 (6.10) |
| 15. Gender (P) |  |  |  |  |  |  |  |  |  |  |  |  |  |  | .01 | -.01 |  |
| 16. Economic status (P) |  |  |  |  |  |  |  |  |  |  |  |  |  |  |  | .30 | 3.26 (0.84) |
| 17. Education (P) |  |  |  |  |  |  |  |  |  |  |  |  |  |  |  |  | 3.67 (1.27) |

Note: Correlations coefficient values > .07 were significant at p < .05. Correlations coefficient values > .09 were significant at p < .01. In case the same variables were measured in parent and child, intraclass correlation coefficients are provided (see coefficients marked in bold font). ICC values > .15 were significant at p < .01. ICC values > .18 were significant at p < .001. In other cases, Pearson *r* coefficient is reported. T1 – Time 1, baseline; T2 – Time 2, 7-8 -month follow-up. Ch – children data, P – parental data. PA promotion – Parental perceptions of school or community-based PA promotion programs, Transportation – Parental or child perceptions of parental support for child PA (transportation provision).

**Findings from additional two-group model analyses**

We investigated if patterns of associations observed in the total sample are similar to the patterns of associations observed across subgroups of dyads: (1) mother-child dyads in comparison to father-child dyads; (2) parent-daughter dyads in comparison to parent-son dyads and (3) dyads with a parents with normal body weight in comparison to dyads with parents with overweight/obesity.

Overall, all analyses revealed patterns of associations similar to those observed in the total sample. Child MVPA (T2) mediated the relationship between parental perceptions of school or community-based PA promotion programs (T1) and child BMI z-scores (T2). Furthermore, child MVPA (T2) mediated the relationship between parental perceptions of parental transportation provision (T1) and child BMI z-scores (T2). There were no indirect effects of child perceptions of parental transportation provision (T1) on child BMI z-scores (T2), with child MVPA (T2) operating as the mediator.

**Results of path analysis for mother-child dyads in comparison to father-child dyads**

The analysis for the unconstrained two-group model with subgroups of mother-child dyads (*n* = 732) and father-child dyads (*n* = 147) yielded following model-data fit, with χ^2^ (102) = 233.482, *p* < .001, χ^2^/df = 2.289, TLI = .915, NFI = .920, CFI = .952, RMSEA = .038 (90% CI: .032, .045). The unconstrained hypothesized model was then compared with the constrained nested model, assuming that the effects of key study variables are similar, regardless of parental gender. The following paths were assumed to be equal across the two types of dyads: paths from the parental and child perceptions of transportation support and PA promotion (T1) to MVPA (T2) and paths from MVPA (T2) to z-BMI scores (T2). Analysis conducted for this nested model yielded following model-data fit, with χ^2^ (102) = 233.482, *p* < .001, χ^2^/df = 2.229, TLI = .919, NFI = .919, CFI = .953, RMSEA = .037 (90% CI: .031, .044). The comparison between the unconstrained model and the nested model showed that the fit of the two compared models did not differ significantly (ΔTLI of -.004, *p* = .600 for Δχ²). Therefore, the nested model may be accepted. Table 1 displays the values of unstandardized regression and covariance coefficients for the nested model.

The variables in the nested model explained 88% (mother-child dyads) and 90% (fathers-child dyads) of variance of child BMI z-scores (T2). All variables included in the nested two-group model accounted for 14% (mother-child dyads) and 38% (fathers-child dyads) of child MVPA (T2) variance.

In accordance with our main hypothesis, child MVPA (T2) mediated the relationship between parental perceptions of school or community-based PA promotion programs (T1) and child BMI z-scores (T2), with an indirect coefficient (unstandardized value) of B = -0.004, (95% BCI -0.008, -0.001), *p* = 0.019 in the mother-child dyads subgroup, and *B* = -0.004 (95% BCI -0.008, -0.001), *p* = 0.019 in the father-child dyads subgroup.

Furthermore, child MVPA (T2) mediated the relationship between parental perceptions of parental transportation provision (T1) and child BMI z-scores (T2), with an indirect coefficient (unstandardized value) of B = -0.006, (95% BCI -0.012, -0.002), *p* = 0.023 in the mother-child dyads subgroup and *B* = -0.006, (95% BCI -0.012, -0.002), *p* = 0.023 in the father-child dyads subgroup. There were no indirect effects of child perceptions of parental transportation provision (T1) on child BMI z-scores (T2), with child MVPA (T2) operating as the mediator.

Table 1.
*Regression and covariance coefficients for the two-group nested model: mother-child dyads (n=732) and father-child dyads (n=147).*

| Variable | Coefficients in the subsample mother-child dyads | | | Coefficients in the subsample father-child dyads | | | |
| --- | --- | --- | --- | --- | --- | --- | --- |
| Path coefficients/covariance coefficients | Estimate | *S.E.* | *p*-value | Estimate | *S.E.* | *p*-value | |
| **Predictors of child MVPA (T2)** |  |  |  |  |  |  | |
| PA promotion (P, T1) 🡪 MVPA (Ch, T2) | **2.732** | **1.050** | **< .001** | **2.732** | **1.050** | **.009** | |
| Transportation (P, T1) 🡪 MVPA (Ch, T2) | **4.447** | **0.951** | **< .001** | **4.447** | **0.951** | **< .001** | |
| Transportation (Ch, T1) 🡪 MVPA (Ch, T2) | 0.052 | 0.794 | .948 | 0.052 | 0.794 | .948 | |
| BMI (P, T1) 🡪 MVPA (Ch, T2) | 0.273 | 0.233 | .240 | **-**0.600 | 0.497 | .227 | |
| BMI z-scores (Ch, T1) 🡪 MVPA (Ch, T2) | 1.123 | 0.707 | .112 | **4.075** | **1.346** | **.002** | |
| MVPA (P, T1) 🡪 MVPA (Ch, T2) | 0.024 | 0.045 | .590 | 0.014 | 0.091 | .879 | |
| MVPA (Ch, T1) 🡪 MVPA (Ch, T2) | **0.250** | **0.033** | **< .001** | **0.463** | **0.061** | **< .001** | |
| Gender (Ch, T1) 🡪 MVPA (Ch, T2) | -**4.292** | **1.708** | **.012** | **-**1.569 | 3.399 | .644 | |
| Age (P, T1) 🡪 MVPA (Ch, T2) | 0.154 | 0.152 | .309 | 0.241 | 0.240 | .315 | |
| Age (Ch, T1) 🡪 MVPA (Ch, T2) | 1.112 | 0.634 | .079 | -0.968 | 1.335 | .469 | |
| Education (P, T1) 🡪 MVPA (Ch, T2) | **-**0.957 | 0.720 | .184 | **-**1.557 | 1.586 | .326 | |
| Economic status (P, T1) 🡪 MVPA (Ch, T2) | **-**0.769 | 1.124 | .494 | **-**0.017 | 1.816 | .992 | |
| **Predictors of child z-BMI score (T2)** |  |  |  |  |  |  | |
| MVPA (Ch, T2) 🡪 BMI z-scores (Ch, T2) | -**0.001** | **<0.001** | **.021** | **-0.001** | **<0.001** | **.021** | |
| BMI (P, T1) 🡪 BMI z-scores (Ch, T2) | -0.001 | 0.004 | .771 | 0.018 | 0.010 | .080 | |
| BMI z-scores (Ch, T1) 🡪 BMI z-scores (Ch, T2) | **0.935** | **0.013** | **< .001** | **0.957** | **0.027** | **< .001** | |
| Gender (Ch, T1) 🡪 BMI z-scores (Ch, T2) | **-**0.004 | 0.031 | .896 | 0.009 | 0.069 | .899 | |
| Age (P, T1) 🡪 BMI z-scores (Ch, T2) | **-**0.001 | 0.003 | .830 | **-**0.002 | 0.005 | .687 | |
| Age (Ch, T1) 🡪 BMI z-scores (Ch, T2) | **-**0.014 | 0.012 | .242 | 0.016 | 0.037 | .661 | |
| Education (P, T1) 🡪 BMI z-scores (Ch, T2) | **0.035** | **0.013** | **.005** | -0.045 | 0.032 | .157 | |
| Economic status (P, T1) 🡪 BMI z-scores (Ch, T2) | **-**0.012 | 0.020 | .553 | 0.016 | 0.037 | .661 | |
| **Covariances** |  |  |  |  |  |  | |
| PA promotion (P, T1) 🡨🡪 MVPA (Ch, T1) | -0.392 | 0.699 | .575 | 1.413 | 1.823 | .438 | |
| PA promotion (P, T1) 🡨🡪 Transportation (P, T1) | 0.044 | 0.025 | .084 | 0.021 | 0.063 | .743 | |
| PA promotion (P, T1) 🡨🡪 Transportation (Ch, T1) | 0.024 | 0.030 | .408 | 0.040 | 0.072 | .581 | |
| PA promotion (P, T1) 🡨🡪 Economic status (P, T1) | 0.010 | 0.021 | .647 | 0.035 | 0.067 | .601 | |
| PA promotion (P, T1) 🡨🡪 Education (P, T1) | **-**0.031 | 0.034 | .368 | **-**0.131 | 0.078 | .091 | |
| Transportation (P, T1) 🡨🡪 MVPA (Ch, T1) | **2.691** | **0.881** | **.002** | 3.883 | 2.133 | .069 | |
| Transportation (P, T1) 🡨🡪 Transportation (Ch, T1) | **0.492** | **0.043** | **< .001** | **0.573** | **0.099** | **< .001** | |
| Transportation (P, T1) 🡨🡪 Economic status (P, T1) | **0.163** | **0.029** | **< .001** | **0.269** | **0.083** | **< .001** | |
| Transportation (P, T1) 🡨🡪 Education (P, T1) | **0.417** | **0.048** | **< .001** | **0.342** | **0.097** | **< .001** | |
| Transportation (Ch, T1) 🡨🡪 MVPA (Ch, T1) | **5.555** | **1.087** | **< .001** | **8.637** | **2.618** | **< .001** | |
| Transportation (Ch, T1) 🡨🡪 Economic status (P, T1) | **0.133** | **0.033** | **< .001** | **0.179** | **0.090** | **.047** | |
| Transportation (Ch, T1) 🡨🡪 Education (P, T1) | **0.265** | **0.053** | **< .001** | 0.121 | 0.103 | .239 | |
| MVPA (P, T1) 🡨🡪 BMI (P, T1) | -**5.393** | **2.673** | **.004** | **-11.822** | **5.819** | **.042** | |
| MVPA (P, T1) 🡨🡪 BMI z-scores (Ch, T1) | 0.597 | 0.875 | .495 | -3.519 | 2.129 | .098 | |
| MVPA (Ch, T1) 🡨🡪 MVPA (P, T1) | **92.833** | **19.096** | **< .001** | **128.069** | **46.154** | **.006** | |
| MVPA (Ch, T1) 🡨🡪 BMI (P, T1) | 0.307 | 3.652 | .933 | **-**1.854 | 8.185 | .821 | |
| MVPA (Ch, T1) 🡨🡪 BMI z-scores (Ch, T1) | **-**0.408 | 1.199 | .733 | 0.903 | 3.010 | .764 | |
| BMI (P, T1) 🡨🡪 BMI z-scores (Ch, T1) | **0.868** | **0.173** | **< .001** | **1.090** | **0.395** | **.006** | |
| BMI (P, T1) 🡨🡪 Age (P, T1) | **3.813** | **0.800** | **< .001** | 3.261 | 2.059 | | .113 |
| Economic status (P, T1) 🡨🡪 Education (P, T1) | **0.295** | **0.040** | **< .001** | **0.466** | **0.106** | **< .001** | |

*Note*: MVPA – Moderate to vigorous physical activity, P – parent, Ch – child, T1 – Time 1, T2 – Time 2 (7-8-month follow-up), PA promotion – Parental perceptions of school or community-based PA promotion programs, Transportation – Parental or child perceptions of parental support for child PA (transportation provision). Significant coefficients are marked in bold.

**Results of path analysis for parent-daughter dyads in comparison to parent-son dyads**

Analysis for the unconstrained two-group model with subgroups of parent-daughter dyads (*n* = 461) and parent-son dyads (*n* = 418) yielded following model-data fit, with χ^2^ (104) = 294.531, *p* < .001, χ^2^/df = 2.874, TLI = .881, NFI = .906, CFI = .935, RMSEA = .046 (90% CI: .040, .053). The unconstrained hypothesized model was then compared with the constrained nested model, assuming that the effects of key study variables are similar, regardless of children’s gender. The following paths were assumed to be equal across the two types of dyads: paths from the parental and child perceptions of transportation support and PA promotion (T1) to MVPA (T2) and paths from MVPA (T2) to z-BMI scores (T2). Analysis conducted for this nested model yielded following model-data fit, with χ^2^ (104) = 294.531, *p* < .001, χ^2^/df = 2.832, TLI = .881, NFI = .906, CFI = .935, RMSEA = .046 (90% CI: .040, .052). The comparison between the unconstrained model and the nested model showed that the fit of the two compared models did not differ significantly (ΔTLI of -.003, *p* = .131 for Δχ²). Therefore, the nested model may be accepted. Table 2 displays the values of unstandardized regression and covariance coefficients obtained for the nested model.

The variables in the nested model explained 89% (parent-daughter dyads) and 88% (parent-son dyads) of variance of child BMI z-scores (T2). All variables included in the two-group nested model accounted for 24% (parent-daughter dyads) and 13% (parent-son dyads) of child MVPA (T2) variance.

In accordance with our main hypothesis, child MVPA (T2) mediated the relationship between parental perceptions of school or community-based PA promotion programs (T1) and child BMI z-scores (T2), with an indirect coefficient (unstandardized value) of B = -0.004, (95% BCI -0.009, -0.001), *p* = 0.018 in the parent-daughter dyads subgroup, and *B* = -0.004 (95% BCI -0.009, -0.001), *p* = 0.018 in the parent-son dyads subgroup.

Furthermore, child MVPA (T2) mediated the relationship between parental perceptions of parental transportation provision (T1) and child BMI z-scores (T2), with an indirect coefficient (unstandardized value) of B = -0.005, (95% BCI -0.012, -0.001), *p* = 0.022 in the parent-daughter dyads subgroup and *B* = -0.005, (95% BCI -0.012, -0.001), *p* = 0.022 in the parent-son dyads subgroup. There were no indirect effects of child perceptions of parental transportation provision (T1) on child BMI z-scores (T2), with child MVPA (T2) operating as the mediator.

Table 2.
*Regression and covariance coefficients for the for the two-group nested model: parent-daughter dyads (n=461) and parent-son dyads (n=418).*

| Variable | Coefficients in the subsample parent-daughter dyads | | | Coefficients in the subsample parent-son dyads | | | |
| --- | --- | --- | --- | --- | --- | --- | --- |
| Path coefficients/covariance coefficients | Estimate | *S.E.* | *p*-value | Estimate | *S.E.* | *p*-value | |
| **Predictors of child MVPA (T2)** |  |  |  |  |  |  | |
| PA promotion (P, T1) 🡪 MVPA (Ch, T2) | **2.952** | **1.032** | **.004** | **2.952** | **1.032** | **.004** | |
| Transportation (P, T1) 🡪 MVPA (Ch, T2) | **4.212** | **0.924** | **< .001** | **4.212** | **0.924** | **< .001** | |
| Transportation (Ch, T1) 🡪 MVPA (Ch, T2) | 0.408 | 0.775 | .599 | 0.408 | 0.775 | .599 | |
| BMI (P, T1) 🡪 MVPA (Ch, T2) | 0.004 | 0.261 | .987 | 0.277 | 0.338 | .411 | |
| BMI z-scores (Ch, T1) 🡪 MVPA (Ch, T2) | 1.428 | 0.787 | .070 | 1.901 | 0.989 | .054 | |
| MVPA (P, T1) 🡪 MVPA (Ch, T2) | -0.050 | 0.049 | .306 | 0.116 | 0.066 | .081 | |
| MVPA (Ch, T1) 🡪 MVPA (Ch, T2) | **0.334** | **0.035** | **< .001** | **0.246** | **0.048** | **< .001** | |
| Gender (P, T1) 🡪 MVPA (Ch, T2) | 1.091 | 2.644 | .680 | 3.237 | 3.396 | .341 | |
| Age (P, T1) 🡪 MVPA (Ch, T2) | **0.296** | **0.148** | **.045** | -0.036 | 0.221 | .870 | |
| Age (Ch, T1) 🡪 MVPA (Ch, T2) | **1.521** | **0.703** | **.031** | -0.210 | 0.917 | .819 | |
| Education (P, T1) 🡪 MVPA (Ch, T2) | **-1.809** | **0.770** | **.019** | **-**0.070 | 1.085 | .948 | |
| Economic status (P, T1) 🡪 MVPA (Ch, T2) | 1.321 | 1.160 | .255 | **-3.717** | **1.582** | **.019** | |
| **Predictors of child z-BMI score (T2)** |  |  |  |  |  |  | |
| MVPA (Ch, T2) 🡪 BMI z-scores (Ch, T2) | -**0.001** | **<0.001** | **.032** | -**0.001** | **<0.001** | **.032** | |
| BMI (P, T1) 🡪 BMI z-scores (Ch, T2) | 0.003 | 0.005 | .617 | -0.001 | 0.006 | .905 | |
| BMI z-scores (Ch, T1) 🡪 BMI z-scores (Ch, T2) | **0.946** | **0.016** | **< .001** | **0.930** | **0.017** | **< .001** | |
| Gender (P, T1) 🡪 BMI z-scores (Ch, T2) | 0.040 | 0.054 | .459 | 0.013 | 0.059 | .820 | |
| Age (P, T1) 🡪 BMI z-scores (Ch, T2) | 0.002 | 0.003 | .472 | **-**0.005 | 0.004 | .189 | |
| Age (Ch, T1) 🡪 BMI z-scores (Ch, T2) | **-0.028** | **0.014** | **.048** | 0.011 | 0.016 | .500 | |
| Education (P, T1) 🡪 BMI z-scores (Ch, T2) | 0.025 | 0.015 | .098 | 0.029 | 0.018 | .111 | |
| Economic status (P, T1) 🡪 BMI z-scores (Ch, T2) | **-**0.006 | 0.023 | .801 | -0.014 | 0.027 | .609 | |
| **Covariances** |  |  |  |  |  |  | |
| PA promotion (P, T1) 🡨🡪 MVPA (Ch, T1) | -0.977 | 0.924 | .290 | 0.566 | 0.914 | .536 | |
| PA promotion (P, T1) 🡨🡪 Transportation (P, T1) | 0.025 | 0.033 | .446 | 0.043 | 0.033 | .192 | |
| PA promotion (P, T1) 🡨🡪 Transportation (Ch, T1) | -0.019 | 0.037 | .598 | 0.069 | 0.041 | .091 | |
| PA promotion (P, T1) 🡨🡪 Economic status (P, T1) | 0.002 | 0.029 | .946 | 0.025 | 0.029 | .399 | |
| PA promotion (P, T1) 🡨🡪 Education (P, T1) | **0.304** | **0.054** | **< .001** | **-**0.006 | 0.044 | .886 | |
| Transportation (P, T1) 🡨🡪 MVPA (Ch, T1) | **2.522** | **1.185** | **.033** | **2.627** | **1.074** | **.014** | |
| Transportation (P, T1) 🡨🡪 Transportation (Ch, T1) | **0.476** | **0.054** | **< .001** | **0.521** | **0.057** | **< .001** | |
| Transportation (P, T1) 🡨🡪 Economic status (P, T1) | **0.166** | **0.039** | **< .001** | **0.190** | **0.039** | **< .001** | |
| Transportation (P, T1) 🡨🡪 Education (P, T1) | **0.357** | **0.061** | **< .001** | **0.449** | **0.060** | **< .001** | |
| Transportation (Ch, T1) 🡨🡪 MVPA (Ch, T1) | **4.546** | **1.372** | **< .001** | **7.052** | **1.444** | **< .001** | |
| Transportation (Ch, T1) 🡨🡪 Economic status (P, T1) | **0.145** | **0.043** | **< .001** | **0.130** | **0.045** | **.004** | |
| Transportation (Ch, T1) 🡨🡪 Education (P, T1) | **0.214** | **0.066** | **< .001** | **0.256** | **0.067** | **< .001** | |
| MVPA (P, T1) 🡨🡪 BMI (P, T1) | -5.667 | 3.429 | .098 | **-11.822** | **5.819** | **.042** | |
| MVPA (P, T1) 🡨🡪 BMI z-scores (Ch, T1) | -0.177 | 1.129 | .875 | **-7.831** | **3.430** | **.022** | |
| MVPA (Ch, T1) 🡨🡪 MVPA (P, T1) | **126.844** | **26.199** | **< .001** | **68.285** | **23.346** | **.003** | |
| MVPA (Ch, T1) 🡨🡪 BMI (P, T1) | 2.835 | 4.787 | .554 | **-**1.850 | 4.576 | .686 | |
| MVPA (Ch, T1) 🡨🡪 BMI z-scores (Ch, T1) | **-**0.511 | 1.580 | .747 | -0.207 | 1.552 | .894 | |
| BMI (P, T1) 🡨🡪 BMI z-scores (Ch, T1) | **0.968** | **0.216** | **< .001** | **0.833** | **0.233** | **< .001** | |
| BMI (P, T1) 🡨🡪 Age (P, T1) | **4.578** | **1.132** | **< .001** | **3.238** | **1.022** | | **.002** |
| BMI (P, T1) 🡨🡪 Gender (P, T1) | **-0.298** | **0.064** | **< .001** | **-0.479** | **0.076** | | **< .001** |
| Economic status (P, T1) 🡨🡪 Education (P, T1) | **0.304** | **0.054** | **< .001** | **0.466** | **0.106** | **< .001** | |

*Note*: MVPA – Moderate to vigorous physical activity, P – parent, Ch – child, T1 – Time 1, T2 – Time 2 (7-8-month follow-up), PA promotion – Parental perceptions of school or community-based PA promotion programs, Transportation – Parental or child perceptions of parental support for child PA (transportation provision). Significant coefficients are marked in bold.

**Results of path analysis for dyads with parents with normal body weight in comparison to dyads with parents with overweight/obesity**

Analysis for the unconstrained two-group model with subgroups of parents with normal body weight (*n* =523) and parents with overweight/obesity (*n* = 338) yielded following model-data fit, with χ^2^ (124) = 316.739, *p* < .001, χ^2^/df = 2.554, TLI = .880, NFI = .892, CFI = .929, RMSEA = .043 (90% CI: .037, .048). As in previous analyses, this unconstrained hypothesized model was compared with the constrained nested model, assuming that the effects of key study variables are similar, regardless of parental overweight/obesity status. The following paths were assumed to be equal across the two types of dyads: paths from the parental and child perceptions of transportation support and PA promotion (T1) to MVPA (T2) and paths from MVPA (T2) to z-BMI scores (T2). Analysis conducted for this nested model yielded following model-data fit, with χ^2^ (124) = 316.739, *p* < .001, χ^2^/df = 2.536, TLI = .881, NFI = .889, CFI = .928, RMSEA = .042 (90% CI: .037, .048). The comparison between the unconstrained model and the nested model showed that the fit of the two compared models did not differ significantly (ΔTLI of -.001, *p* = .099 for Δχ²). Thus, the constrained model may be accepted. Table 3 displays the values of unstandardized regression and covariance coefficients obtained for the nested model.

The variables in the hypothesized two-group nested model explained 89% (parents with normal body weight) and 87% (parents with overweight/obesity) of variance of child BMI z-scores (T2). All variables included in the two-group nested model accounted for 18% (parents with normal body weight) and 19% (parents with overweight/obesity) of child MVPA (T2) variance.

In accordance with our main hypothesis, child MVPA (T2) mediated the relationship between parental perceptions of school or community-based PA promotion programs (T1) and child BMI z-scores (T2), with an indirect coefficient (unstandardized value) of B = -0.003 (95% BCI -0.009, 0.000), *p* = 0.031 in the parents with normal body weight subgroup, and *B* = -0.003 (95% BCI -0.009, 0.000), *p* = 0.031 in the parents with overweight/obesity subgroup.

Furthermore, child MVPA (T2) mediated the relationship between parental perceptions of parental transportation provision (T1) and child BMI z-scores (T2), with an indirect coefficient (unstandardized value) of B = -0.006, (95% BCI -0.013, 0.000), *p* = 0.039 in the parents with normal body weight dyads subgroup and *B* = -0.006, (95% BCI -0.013, 0.000), *p* = 0.039 in the parents with overweight/obesity subgroup. There were no indirect effects of child perceptions of parental transportation provision (T1) on child BMI z-scores (T2), with child MVPA (T2) operating as the mediator.

Table 3.
*Regression and covariance coefficients for the for the two-group nested model: parents with normal body weight dyads (n=523) and parents with overweight/obesity (n=338).*

| Variable | Coefficients in the subsample parents with normal body weight | | | Coefficients in a subsample parents with overweight/obesity | | | |
| --- | --- | --- | --- | --- | --- | --- | --- |
| Path coefficients/covariance coefficients | Estimate | *S.E.* | *p*-value | Estimate | *S.E.* | *p*-value | |
| **Predictors of child MVPA (T2)** |  |  |  |  |  |  | |
| PA promotion (P, T1) 🡪 MVPA (Ch, T2) | **2.656** | **1.072** | **.013** | **2.656** | **1.072** | **.013** | |
| Transportation (P, T1) 🡪 MVPA (Ch, T2) | **4.537** | **0.967** | **< .001** | **4.537** | **0.967** | **< .001** | |
| Transportation (Ch, T1) 🡪 MVPA (Ch, T2) | 0.203 | 0.802 | .801 | 0.203 | 0.802 | .801 | |
| BMI (P, T1) 🡪 MVPA (Ch, T2) | **1.533** | **0.580** | **.008** | 0.087 | 0.404 | .830 | |
| BMI z-scores (Ch, T1) 🡪 MVPA (Ch, T2) | 0.304 | 0.825 | .713 | **3.660** | **1.011** | **< .001** | |
| MVPA (P, T1) 🡪 MVPA (Ch, T2) | -0.001 | 0.054 | .992 | 0.059 | 0.066 | .371 | |
| MVPA (Ch, T1) 🡪 MVPA (Ch, T2) | **0.326** | **0.038** | **< .001** | **0.235** | **0.046** | **< .001** | |
| Gender (P, T1) 🡪 MVPA (Ch, T2) | 2.945 | 4.125 | .475 | 1.426 | 2.563 | .578 | |
| Gender (Ch, T1) 🡪 MVPA (Ch, T2) | -1.623 | 2.030 | .424 | **-6.676** | **2.413** | **.006** | |
| Age (P, T1) 🡪 MVPA (Ch, T2) | 0.094 | 0.192 | .624 | 0.260 | 0.174 | .135 | |
| Age (Ch, T1) 🡪 MVPA (Ch, T2) | 0.465 | 0.743 | .532 | 1.348 | 0.949 | .156 | |
| Education (P, T1) 🡪 MVPA (Ch, T2) | -0.823 | 0.844 | .330 | **-2.236** | **1.050** | **.033** | |
| Economic status (P, T1) 🡪 MVPA (Ch, T2) | -0.625 | 1.302 | .631 | -0.306 | 1.462 | .834 | |
| **Predictors of child z-BMI score (T2)** |  |  |  |  |  |  | |
| MVPA (Ch, T2) 🡪 BMI z-scores (Ch, T2) | -**0.001** | **<0.001** | **.033** | -**0.001** | **<0.001** | **.033** | |
| BMI (P, T1) 🡪 BMI z-scores (Ch, T2) | 0.017 | 0.010 | .105 | -0.002 | 0.008 | .751 | |
| BMI z-scores (Ch, T1) 🡪 BMI z-scores (Ch, T2) | **0.926** | **0.015** | **< .001** | **0.956** | **0.020** | **< .001** | |
| Gender (P, T1) 🡪 BMI z-scores (Ch, T2) | **0.150** | **0.074** | **.042** | -0.025 | 0.051 | .621 | |
| Gender (Ch, T1) 🡪 BMI z-scores (Ch, T2) | -0.030 | 0.036 | .408 | 0.019 | 0.048 | .688 | |
| Age (P, T1) 🡪 BMI z-scores (Ch, T2) | 0.001 | 0.003 | .813 | **-**0.002 | 0.003 | .654 | |
| Age (Ch, T1) 🡪 BMI z-scores (Ch, T2) | -0.006 | 0.013 | .645 | -0.027 | 0.019 | .148 | |
| Education (P, T1) 🡪 BMI z-scores (Ch, T2) | 0.020 | 0.015 | .168 | 0.032 | 0.020 | .112 | |
| Economic status (P, T1) 🡪 BMI z-scores (Ch, T2) | **-**0.004 | 0.023 | .878 | -0.025 | 0.029 | .384 | |
| **Covariances** |  |  |  |  |  |  | |
| PA promotion (P, T1) 🡨🡪 MVPA (Ch, T1) | 0.144 | 0.842 | .864 | -0.744 | 1.063 | .484 | |
| PA promotion (P, T1) 🡨🡪 Transportation (P, T1) | **0.080** | **0.031** | **.009** | -0.023 | 0.039 | .558 | |
| PA promotion (P, T1) 🡨🡪 Transportation (Ch, T1) | 0.020 | 0.036 | .565 | 0.042 | 0.045 | .347 | |
| PA promotion (P, T1) 🡨🡪 Economic status (P, T1) | 0.022 | 0.026 | .384 | 0.003 | 0.036 | .922 | |
| PA promotion (P, T1) 🡨🡪 Education (P, T1) | -0.004 | 0.040 | .911 | **-0.127** | **0.051** | **.013** | |
| Transportation (P, T1) 🡨🡪 MVPA (Ch, T1) | **2.813** | **1.074** | **.009** | **2.553** | **1.280** | **.046** | |
| Transportation (P, T1) 🡨🡪 Transportation (Ch, T1) | **0.504** | **0.052** | **< .001** | **0.520** | **0.064** | **< .001** | |
| Transportation (P, T1) 🡨🡪 Economic status (P, T1) | **0.154** | **0.035** | **< .001** | **0.215** | **0.047** | **< .001** | |
| Transportation (P, T1) 🡨🡪 Education (P, T1) | **0.270** | **0.063** | **< .001** | **0.467** | **0.070** | **< .001** | |
| Transportation (Ch, T1) 🡨🡪 MVPA (Ch, T1) | **6.295** | **1.314** | **< .001** | **5.066** | **1.605** | **.002** | |
| Transportation (Ch, T1) 🡨🡪 Economic status (P, T1) | **0.159** | **0.040** | **< .001** | **0.110** | **0.053** | **.036** | |
| Transportation (Ch, T1) 🡨🡪 Education (P, T1) | **0.270** | **0.063** | **< .001** | **0.186** | **0.075** | **.013** | |
| MVPA (P, T1) 🡨🡪 BMI (P, T1) | 0.392 | 1.490 | .792 | **-9.385** | **3.215** | **.004** | |
| MVPA (P, T1) 🡨🡪 BMI z-scores (Ch, T1) | 1.064 | 1.050 | .311 | -1.982 | 1.257 | .115 | |
| MVPA (Ch, T1) 🡨🡪 MVPA (P, T1) | **91.063** | **22.678** | **< .001** | **118.305** | **28.877** | **< .001** | |
| MVPA (Ch, T1) 🡨🡪 BMI (P, T1) | -0.933 | 2.059 | .650 | 2.644 | 4.468 | .554 | |
| MVPA (Ch, T1) 🡨🡪 BMI z-scores (Ch, T1) | -0.979 | 1.450 | .499 | 0.649 | 1.763 | .713 | |
| BMI (P, T1) 🡨🡪 BMI z-scores (Ch, T1) | **0.381** | **0.098** | **< .001** | 0.240 | 0.199 | .228 | |
| BMI (P, T1) 🡨🡪 Age (P, T1) | **0.913** | **0.409** | **.025** | **2.925** | **1.164** | | **.012** |
| BMI (P, T1) 🡨🡪 Gender (P, T1) | **-0.045** | **0.019** | **.018** | **0.015** | **0.077** | | **.841** |
| Economic status (P, T1) 🡨🡪 Education (P, T1) | **0.289** | **0.047** | **< .001** | **0.381** | **0.064** | **< .001** | |

*Note*: MVPA – Moderate to vigorous physical activity, P – parent, Ch – child, T1 – Time 1, T2 – Time 2 (7-8-month follow-up), PA promotion – Parental perceptions of school or community-based PA promotion programs, Transportation – Parental or child perceptions of parental support for child PA (transportation provision). Significant coefficients are marked in bold.
